# Assessment of stress level and depression among orthopaedic surgeons in Saudi Arabia

**DOI:** 10.1007/s00264-024-06288-0

**Published:** 2024-09-05

**Authors:** Wazzan S. Aljuhani, Ziad A. Aljaafri, Khalid H. Alhadlaq, Abdullah M. Alanazi, Abdulrahman K. Alhadlaq, Meshal K. Alaqeel

**Affiliations:** 1 Department of Orthopedic Surgery, Ministry of the National Guard – Health Affairs, Riyadh, Saudi Arabia; 2 King Abdullah International Medical Research Center, Riyadh, Saudi Arabia; 3 King Saud bin Abdulaziz University for Health Sciences, Riyadh, Saudi Arabia; 4 Department of Psychiatry, Ministry of the National Guard – Health Affairs, Riyadh, Saudi Arabia

**Keywords:** Stress level, Depression, Orthopaedic surgery, Burnout, Saudi Arabia

## Abstract

**Purpose:**

The aim of this study was to assess the stress level and depression among orthopaedic surgeons in Saudi Arabia. In addition, to evaluate orthopedic training programs related factors that might have a critical role in the development of depression among orthopaedic surgeons.

**Methods:**

The study adopted a cross-sectional study design. Two validated questionnaires were utilized, the Patient Health Questionnaire 9 (PHQ-9) and the Perceived Stress Scale (PSS-10) for assessing depressive symptoms and stress levels. Data was collected by sending the survey to the Saudi Commission for Health Specialties so they could be distributed throughout all registered orthopaedic surgeons.

**Results:**

The study sample consisted of 325 participants. The results revealed that the severity of depression varied across the different groups. As per the PHQ-9 criteria, 74 (22.8%) were initially diagnosed with major depression. Among assistant consultants, 39.5% reported severe depression, while 34.9% reported mild depression. Consultants predominantly reported moderate perceived stress (82.9%) with a notable proportion experiencing high perceived stress (12.4%). Assistant consultants showed a balanced distribution, with 93.0% reporting moderate perceived stress and 4.7% reporting high perceived stress. Demographic variables gender, relationship status and having children revealed statistically significant relationship with PHQ-9 scores (p-value < 0.05) but not with PSS-10 scores.

**Conclusion:**

The study highlights pressing need to address mental health concerns within orthopaedic surgeons. To address these challenges, healthcare institutions should implement comprehensive mental health support programs offering resources for stress management, counseling services, and peer support groups.

## Introduction

Burnout and depression have recently been looked at differently due to its critical role and influence that it might have, not only on physicians themselves but also the quality of care provided to the patients [1, 4]. At time of increasing risk of suicidal thoughts and burnout among health care personnel [1]. Screening for burnout and depression among health care workers has recently received more attention lately. Surgical training, in comparison to medical training, has long been regarded as demanding, but its unique mental health challenges are rarely addressed. In addition to having negative psychological and physical effects on individuals, depression has been linked to decreased patient care and an increase in medical errors [2, 3].

There is increased risk of burnout and depression among medical personnel at different levels compared to their peers in non-medical field [5]. All specialties are affected even though they may vary in extinct based on level of training, gender, or the presence of social and personal support [10]. However, for orthopaedic surgery residents there was only one study conducted that looked at the burnout and quality of life [6], other studied the mental health among orthopedic residents [7]. Recent study showed that burnout among orthopaedic surgery residents reached 52% in addition to depression which was found to be in 13% [8]. In addition, stress which is a situation that disturbs or is likely to disturb a person’s normal psychological or physiological functioning [10]. Considered to be an important issue that have to be studied among medical personnel, one study conducted recently showed that around 80% of the doctors had moderate level of stress [11].

Using the patient health questionnaire-9 (PHQ-9) which is a 9-questions survey which has been validated and wildly used to assess for depression among participants, it has been reliable and accepted as well [9, 12, 13]. Moreover, Utilizing the perceived stress scale-10 (PSS-10) have been used to evaluate the stress level, a 10-questions survey that have been used in multiple studies to assess perception of stress [14].

Limited number of studies looked at the mental health in terms of stress level and depression among orthopaedic surgeons internationally, no studies conducted locally. Therefore, we aimed to assess the stress level and depression among orthopaedic surgeons in Saudi Arabia.

## Materials and methods

A cross sectional study was carried out across Saudi Arabia to assess the depression and stress level among orthopaedic surgeons at different levels, by sending a questionnaire that was prepared by the research team to the Saudi Commission for Health Specialties (SCFHS).

The questionnaire prepared included four main parts, participants’ demographics, program characteristics, and the two validated tests utilized in the study including patient health questionnaire-9 (PHQ-9) and the perceived stress scale-10 (PSS-10). The scoring of items in the PHQ-9 ranges from “0” (not at all) to “3” (nearly everyday). Moreover, a total PHQ-9 score of 0 to 4 points is considered “normal” or “minimal” depression. A score of 5 to 9 indicates mild depression, 10 to 14 for moderate depression, 15 to 19 for moderately severe depression, and 20 or more for severe depression [13, 15]. Regarding the scoring of items in the PSS-10 ranges from “0” (never) to “4” (very often). Furthermore, a total of PSS-10 score of 0 to 13 points is considered “low stress”, a score of 14 to 26 points is considered “moderate stress”, and a score of 27 to 40 is considered “high stress”.

Initial diagnosis of major depressive disorder could be made if the following criteria were met in the PHQ-9, five questions or more are checked as more than half the days, and either questions 1 or 2 should be checked with at least more than half the days.

All data collected from Microsoft Excel sheet was transferred to statistical software SPSS file for analysis. Data were checked for any missing information and new variables were recorded and computed based on the data extracted. The categorical data were presented by percentages and frequencies such as gender. Whereas the numerical data were prescribed as mean and standard deviation such as age. For inferential statistics, Chi square was used to find the association between the categorical variable. The test was considered significant if p-value is less than 0.05.

### Ethical approval

was obtained by King Abdullah International Medical Research Center (KAIMRC). Consent was obtained from participants before being involved in this cross-sectional study. All data were kept confidential with no identifications being asked or presented. The privacy of all participants was assured, and access to research data was limited to the study group members.

## Results

Table 1 below represents a demographic characteristic of the participants of the study where an overall of 325 surgeons participated in this research. Among the surgeons the average age and standard deviation was 40.0 ± 10.8. Moreover, 295 (90.8%) of the surgeons were male and 30 (9.2%) were females. In addition, the average and the standard deviation of BMI of the participants was 28.67 ± 4.6. 240 (73.8%) of the surgeons had children while 85 (26.2%) did not have children. 261 (80.3%) of the participants were married, 59 (18.2%) were single and 5(1.5%) were divorced. On the other hand, 254 (78.2%) of the participants lived with the family, 66 (20.3%) of the participants lived alone and 5 (1.5%) lived with friends. 220 (67.7%) of the participants were smokers 105 (32.3%) were not smokers. Work center distribution shows that the majority of respondents work in the Ministry of Health (43.4%), followed by private hospitals (29.5%), and university hospitals (6.2%). Finally, the regional distribution of respondents indicates a diverse geographic spread, with the central region having the highest representation at 28.9%, followed closely by the western region at 28.6%.

**Table 1:** Demographic characteristics of the participants (*N* = 325)

Continuous Variables	Mean/ Std. Deviation	
Age	40. 0 ± 10.8	
BMI	28.67 ± 4.6	
**Categorical Variables**	**Category**	**Count (%)**
Gender	Male	295 (90.8%)
	Female	30 (9.2%)
Relationship Status	Single	59 (18.2%)
	Married	261 (80.3%)
	Divorced	5(1.5%)
Children	Yes	240 (73.8%)
	No	85 (26.2%)
Living Status	Alone	66 (20.3%)
	With Family	254 (78.2%)
	With Friends	5 (1.5%)
Smoking	Yes	220 (67.7%)
	No	105 (32.3%)
Work Center	Military Hospital	31(9.5%)
	Ministry of Health	141(43.4%)
	National Guard	9(2.8%)
	Private Hospital	96(29.5%)
	Security Forces Hospital	4(1.2%)
	Specialist Hospital	24(7.4%)
	University Hospital	20(6.2%)
Region	Central Region	94(28.9%)
	Eastern Region	74(22.8%)
	Northern Region	21(6.5%)
	Southern Region	43(13.2%)
	Western Region	93(28.6%)

Categorical demographic information presented in frequencies (n) and proportion (%). Continuous variables presented mean ± standard deviation

Table 2 bellow presents the program characteristics responses of the individuals that participated on the stress assessment and depression among the orthopaedic surgeons in Saudi Arabia. Majority 110 (33.8%) agreed that surgical independence was excellent while 15 (4.6%) of the surgeons said surgical independence was poor. Most 120 (36.9%) of the surgeons claimed that the faculties reputation was very good while 13 (4.0%) claimed that it was poor. Majority 112 (34.5%) reported that the volume of cases was very good while 11 (3.4%) reported that they were poor. 93 (28.6%) of the surgeons indicated that variety of cases was good while only 13 (4.0%) indicated they were poor. Majority 107 (32.9%) reported that educational support was poor while 26 (8.0%) indicated they were excellent. In addition, 96 (29.5%) indicated that the satisfaction level was good. Also, 114 (35.1%) indicated that their quality of life was good. Similarly, majority 118 (36.3%) of the surgeons reported that the on-call amount was good.

**Table 2:** Program characteristics

Responses	Excellent	Very Good	Good	Fair	Poor
Surgical Independence	110 (33.8%)	85 (26.2%)	86 (26.5%)	29 (8.9%)	15 (4.6%)
Faculties Reputation	90 (27.7%)	120 (36.9%)	72 (22.2%)	30 (9.2%)	13 (4.0%)
Volume of Cases	72 (22.2%)	112 (34.5%)	97 (29.8%)	33 (10.2%)	11(3.4%)
Variety of Cases	70 (21.5%)	116 (35.7%)	93 (28.6%)	33 (10.2%)	13 (4.0%)
Educational Support	26 (8.0%)	55 (16.9%)	74 (22.8%)	63 (19.4%)	107 (32.9%)
Satisfaction	25 (7.7%)	77 (23.7%)	96 (29.5%)	78 (24.0%)	49 (15.1%)
Quality of Life	25 (7.7%)	83 (25.5%)	114 (35.1%)	62 (19.1%)	41 (12.6%)
On-calls Amount	18 (5.5%)	60 (18.5%)	118 (36.3%)	66 (20.3%)	63 (19.4%)

Program characteristics information presented in frequencies (n) and proportion (%)

Table 3 gives an illustration on the patient health questionnaire. Majority 147 (45.2%) showed little interest or pleasure in doing things in several days while 41 (12.6%) showed little interest or pleasure in doing things in nearly every day. Most 142 (43.7%) of participant felt down, depressed or hopeless in several days, 40 (12.3%) showed similar characteristics in nearly every day. Majority 110 (33.8%) did not at all experience trouble in falling or staying asleep or sleeping too much. Most 134 (41.2%) felt tired or had little energy in several days, 53 (16.3%) had trouble falling or staying asleep or sleeping too much in nearly every day. 120 (36.9%) did not have poor appetite or overeating, 43 (14.8%) had poor appetite or overeating nearly every day. Majority 137 (42.2%) did not at all feel bad about themselves or that seeing themselves as failures or letting their family down. 118 (36.3%) did not at all got Trouble in concentrating on things, such as reading the newspaper or watching television. More than a half 194 (59.7%) did not at all move or speaking so slowly that other people could have noticed or the opposite. A super majority 257 (79.1%) did not at all have thoughts that they would be better off dead or of hurting themselves in some way.

**Table 3:** Patient health questionnaire − 9

PHQ-9 Response	Not at all	Several Days	More than half the Days	Nearly Every Day
1. Little interest or pleasure in doing things	73 (22.5%)	147 (45.2%)	64 (19.7%)	41 (12.6%)
2. Feeling down, depressed or hopeless	82 (25.2%)	142 (43.7%)	61 (18.8%)	40 (12.3%)
3. Trouble falling or staying asleep or sleeping too much	110 (33.8%)	106 (32.6%)	56 (17.2%)	53 (16.3%)
4. Feeling tired or having little energy	51 (15.7%)	134 (41.2%)	78 (24.0%)	62 (19.1%)
5. Poor appetite or overeating	120 (36.9%)	103 (31.7%)	54 (16.6%)	48 (14.8%)
6. Feeling bad about yourself — or that you are a failure or have let yourself or your family down	137 (42.2%)	96 (29.5%)	47 (14.5%)	45 (13.8%)
7. Trouble concentrating on things, such as reading the newspaper or watching television	118 (36.3%)	109 (33.5%)	53 (16.3%)	45 (13.8%)
8. Moving or speaking so slowly that other people could have noticed? Or the opposite — being so fidgety or restless that you have been moving around a lot more than usual	194 (59.7%)	77 (23.7%)	36 (11.1%)	18 (5.5%)
9. Thoughts that you would be better off dead or of hurting yourself in some way	257 (79.1%)	44 (13.5%)	11 (3.4%)	13 (4.0%)

Program characteristics information presented in frequencies (n) and proportion (%)

The Table (4) provided depicts the depression levels among medical professionals across different training levels. Among the total respondents, the majority were consultants, totaling 105, followed by residents with 99 respondents, and assistant consultants with 43 respondents. The severity of depression varied across the different groups. Among assistant consultants, 39.5% reported severe depression, while 34.9% reported mild depression. Similarly, among associate consultants, 40.0% reported mild depression, and 26.7% reported moderate depression. Notably, among fellows, the distribution was more evenly spread, with 31.3% reporting mild depression and 25.0% reporting moderately severe depression. Among residents (R1-R5), the prevalence of depression varied, with R2 residents having the highest percentage of mild depression at 38.9%, while 50% of R3 residents reported the highest percentage of moderately severe to severe depression and R4 residents having the highest percentage of moderate to moderately severe depression at 60.0%.

**Table 4:** PHQ-9 score distribution among different training level

Training Level	Depressive Symptoms					Total	Depression	
	**None**	**Mild**	**Moderate**	**Moderately severe**	**Severe**		Yes	No
Assistant Consultant	17(39.5%)	15(34.9%)	5(11.6%)	3(7.0%)	3(7.0%)	43	74 (22.8%)	251 (77.2%)
Associate Consultant	7(23.3%)	12(40.0%)	8(26.7%)	2(6.7%)	1(3.3%)	30		
Board Certified	9(28.1%)	11(34.4%)	7(21.9%)	4(12.5%)	1(3.1%)	32		
Consultant	34(32.4%)	28(26.7%)	25(23.8%)	11(10.5%)	7(6.7%)	105		
Fellow	3(18.8%)	5(31.3%)	3(18.8%)	4(25.0%)	1(6.3%)	16		
R1	12(35.3%)	10(29.4%)	6(17.6%)	3(8.8%) `	3(8.8%)	34		
R2	1(5.6%)	7(38.9%)	3(16.7%)	4(22.2%)	3(16.7%)	18		
R3	1(6.3%)	6(37.5%)	1(6.3%)	4(25.0%)	4(25.0%)	16		
R4	0(0.0%)	2(20.0%)	4(40.0%)	2(20.0%)	2(20.0%)	10		
R5	3(14.3%)	6(28.6%)	7(33.3%)	2(9.5%)	3(14.3%)	21		
Total	87	102	69	39	28	325		

PHQ-9 Score distribution information presented in frequencies (n) and proportion (%)

Table 5 presents the response of the participants on the perceived stressed scale-10. More than half 163 (50.2%) have been sometimes upset because of something that happened unexpectedly, 23 (7.1%) were never upset. Majority122 (37.5%) almost never felt unable to control the important things in your life. Most 136 (41.8%) of the participants in the study sometimes in the past month felt nervous or stressed. 112 (34.5%) of the surgeons in the past month fairly often felt confident about their ability to handle personal problems. 151 (46.5%) of the participants sometimes in the past month, often felt that things were going their way. 134 (41.2%) sometimes in the past month often had been able to control irritations in their life. Majority 142 (43.7%) sometimes in the past month, often felt that they were on top of things. Majority 151 (46.5%) sometimes in the last month, often had been angered because of things that were outside their control. 127 (39.1%) of participants sometimes in the past month they often felt that difficulties were piling up so high that you could not overcome them.

**Table 5:** Perceived stressed scale-10 (PSS-10)

Responses	Never	Almost Never	Sometimes	Fairly Often	Very Often
1. In the past month, how often have you been upset because of something that happened unexpectedly?	23 (7.1%)	51 (15.7%)	163 (50.2%)	55 (16.9%)	33 (10.2%)
2. In the past month, how often have you felt unable to control the important things in your life?	63 (19.4%)	68 (20.9%)	122 (37.5%)	44 (13.5%)	28 (8.6%)
3. In the past month, how often have you felt nervous or stressed?	18 (5.5%)	36 (11.1%)	136 (41.8%)	67 (20.6%)	68 (20.9%)
4. In the past month, how often have you felt confident about your ability to handle personal problems?	18 (5.5%)	26 (8.0%)	97 (29.8%)	112 (34.5%)	72 (22.2%)
5. In the past month, how often have you felt that things were going your way?	25 (7.4%)	38 (11.7%)	151 (46.5%)	79 (24.3%)	33 (10.2%)
6. In the past month, how often have you found that you could not cope with all the things you had to do?	40 (12.3%)	68 (20.9%)	158 (48.6%)	38 (11.7%)	21 (6.5%)
7. In the past month, how often have you been able to control irritations in your life?	11 (3.4%)	38 (11.7%)	134 (41.2%)	90 (27.7%)	52 (16%)
8. In the past month, how often have you felt that you were on top of things?	19(5.8%)	52 (16.0%)	142 (43.7%)	84 (25.8%)	28 (8.6%)
9. In the last month, how often have you been angered because of things that were outside your control?	26(8.0%)	55 (16.9%)	151 (46.5%)	60 (18.5%)	33 (10.2%)
10. In the past month, how often have you felt that difficulties were piling up so high that you could not overcome them?	44 (13.5%)	85 (26.2%)	127 (39.1%)	39 (12.0%)	30 (9.2%)

Perceived stressed scale-10 (PSS-10) presented in frequencies (n) and proportion (%)

The Table (6) below shows the stress level of the 325 individuals that participated in the study. Among consultants, 82.9% reported moderate perceived stress, while 12.4% reported high perceived stress. Assistant consultants, while fewer in number, demonstrated a relatively balanced distribution, with 93.0% reporting moderate perceived stress and 4.7% reporting high perceived stress. Notably, fellows exhibited a higher prevalence of moderate perceived stress at 68.8%, with 18.8% reporting high perceived stress. Among residents, particularly R1 and R2, there was a noticeable trend towards higher levels of perceived stress, with 70.6% and 83.3% reporting moderate perceived stress, respectively. Additionally, 17.6% of R1 residents reported low perceived stress, indicating less responsibility and challenges at the early stages of their training. Among all orthopedic surgeons that participated in the study, a high percentage of surgeons reported having moderate perceived stress at 81.2%, with 6.1% reported having low perceived stress and 12.6% reported having high perceived stress. Indicating an increased stress demand among other specialties at all levels.

**Table 6:** PSS-10 score distribution among the orthopedic surgeons in different training level

Training Level	The level of stress			Total
	Low perceived stress	Moderate perceived stress	High perceived stress	
Assistant Consultant	1(2.3%)	40(93.0%)	2(4.7%)	43
Associate Consultant	1(3.3%)	25(83.3%)	4(13.3%)	30
Board Certified	3(9.4%)	26(81.3%)	3(9.4%)	32
Consultant	5(4.8%)	87(82.9%)	13(12.4%)	105
Fellow	2(12.5%)	11(68.8%)	3(18.8%)	16
R1	6(17.6%)	24(70.6%)	4(11.8%)	34
R2	0(0.0%)	15(83.3%)	3(16.7%)	18
R3	0(0.0%)	12(75.0%)	4(25.0%)	16
R4	0(0.0%)	6(60.0%)	4(40.0%)	10
R5	2(9.5%)	18(85.7%)	1(4.8%)	21
Total	20	264	41	325

PSS-10 Score distribution information presented in frequencies (n) and proportion (%)

Table 7 below shows that females had a significantly higher mean PHQ-9 score compared to males (13.8 vs. 8.7), indicating a higher level of depressive symptoms. Participants who were single had a significantly higher mean PHQ-9 score compared to those who were married or divorced. Participants with children had a significantly lower mean PHQ-9 score compared to those without children. In summary, gender, relationship status, having children, number of children, living status, and smoking status were associated with differences in depressive symptoms (PHQ-9 scores), but not perceived stress (PSS-10 scores).

**Table 7:** Association of stress and depression scores and demographic characteristics

Variable	Category	Mean PHQ-9	*P*-value	Mean PSS-10	*P*-Value
**Gender**	Male	8.7	< 0.001	20.9	0.543
	Female	13.8		23.2	
**Relationship Status**	Single	12.4	< 0.001	22.4	0.442
	Married	8.4		20.8	
**Children**	Yes No	8.2	< 0.001	20.6	0.148
		11.9		22.8	
**Number of Children**	0	11.9	< 0.001	22.8	0.234
	1	8.1		18.5	
	2 to 4	8.3		19.9	
	More than 4	7		20	
**Living Status**	Alone	9.7	< 0.001	22.4	0.463
	With Family	8.9		20.8	
	With Friends	12.8		22.8	
**Smoking**	Yes	8.1	< 0.001	20.5	0.566
	No	11.4		22	

Chi-square test used to determine significance. P-value considered statistically significant at p-value < 0.05

## Discussion

The study aimed to access the stress level and depression among orthopaedic surgeons in Saudi Arabia. The study examined important aspects like the demographic variables and program characteristics to determine potential correlation with perceived stress or depressive symptoms. Most surgeons in the study give positive feedback regarding the program characteristics with 36.9% of surgeons rating the reputation of their faculties as very good, with 27.7% rating it as excellent. Additionally, 35.1% of the surgeons reported very good satisfaction levels, 29.5% rated their quality of life as good. According to a similar study conducted among surgeons in Pakistan the program characteristics was a significant stressor with surgeons working in reputable faculties have low levels of stress or depression [16].

The PHQ-9 responses provide valuable insights into the prevalence of depressive symptoms among surgeons. A significant portion of the surgeons reported experiencing little interest or pleasure in doing things, with 45.2% experiencing this symptom several days and 19.7% experiencing it more than half the days. Similarly, feelings of depression or hopelessness were prevalent, with 43.7% experiencing them several days and 18.8% experiencing them more than half the days. Sleep disturbances were also common, with 33.8% reporting trouble falling or staying asleep or sleeping too much, and 32.6% experiencing this symptom several days. Feelings of tiredness or low energy were reported by 41.2% of surgeons several days and 24.0% more than half the days. In a study that aimed to check the level of depression among high students in Florida, the feeling of being sad or helpless was quite prevalent with about 28% of the students with about 9.6% contemplating suicide [17]. This comparison highlights the widespread nature of depressive symptoms across different demographics.

The distribution of PHQ-9 scores among orthopaedic surgeons reveals a concerning prevalence of depressive symptoms within the population. The data reveals that most consultants experienced either none or mild levels of depression, comprising 32.4% and 26.7% of the group, respectively. However, there were still significant proportions reporting moderate (23.8%) and moderately severe (10.5%) depression. This suggests a substantial portion of consultants facing challenges in managing their mental health. Similarly, associate consultants, assistant consultants, and board-certified professionals also exhibited varying levels of depression across the severity spectrum. While the majority in each group reported none or mild depression, there were notable percentages experiencing moderate to moderately severe depression, emphasizing the prevalence of mental health struggles among these professionals. A study among 467 adult population in Riyadh, Saudi Arabia revealed that 3.6% (17 patients) showed no signs of depression, while 22.1% (103 patients) had minimal depression. Additionally, 34.3% (160 patients) experienced mild depression, 24.2% (113 patients) reported moderate depression, and 10.1% (47 patients) exhibited moderately severe depression. Furthermore, 5.8% (27 patients) were classified as having severe depression [18].

The distribution of PSS-10 scores among orthopaedic surgeons highlights the prevalence of perceived stress within the training levels group. The majority of consultants reported experiencing moderate perceived stress (82.9%), followed by 12.4% reporting high perceived stress and only 4.8% indicating low perceived stress. Similar patterns were observed among assistant consultants, associate consultants, and board-certified professionals, with the majority reporting moderate perceived stress levels. Notably, fellows displayed a higher prevalence of high perceived stress (18.8%) compared to other groups, indicating potential challenges or pressures specific to that stage of their training. Among residents (R1-R5), there was a notable proportion reporting high perceived stress (11.8–25.0%), suggesting the demanding nature of residency programs. Interestingly, while R2 residents showed a high proportion reporting moderate perceived stress (83.3%), there was no reported low perceived stress within this group. In another study conducted by Nazir et al., among doctors revealed the majority of participants, accounting for 79.8%, reported experiencing moderate levels of stress, with 13.8% indicating low stress levels [19].

The study further sort to find the association between stress and depression scores and various demographic characteristics among orthopaedic surgeons. The was gender disparities, with female surgeons exhibiting a significantly higher mean PHQ-9 score (13.8) compared to males (8.7), indicating a greater prevalence of depressive symptoms among female surgeons. Similarly, single surgeons reported a higher mean PHQ-9 score (12.4) compared to their married, or divorced counterparts, suggesting that relationship status may influence depressive symptoms. Furthermore, surgeons without children had a significantly higher mean PHQ-9 score (11.9) compared to those with children, indicating a potential protective effect of parenthood against depression. Additionally, living alone was associated with a higher mean PHQ-9 score (9.7), suggesting that social support and living arrangements may impact depressive symptoms. However, the association between stress scores (PSS-10) and demographic characteristics was not statistically significant. These findings underscore the importance of addressing gender-specific, familial, social, and lifestyle factors in mental health interventions for orthopaedic surgeons, particularly targeting vulnerable subgroups such as female surgeons and those living alone. A study conducted in China among physicians that gender and social support (marriage or staying with family or friends) gave a statistically significant relationship with having perceived stress [20].

A significant limitation of the study stemmed from its use of a cross-sectional study design. While cross-sectional studies excel at identifying relationships between variables at a specific moment, they inherently lack the capacity to establish causality or track changes over time. Therefore, future research efforts should consider employing longitudinal or mixed-methods approaches to provide deeper insights into factors that contribute to stress and depression among surgeons over time.

In conclusion, this study highlights the significant prevalence of perceived stress and depressive symptoms among orthopedic surgeons in Saudi Arabia. The findings from this study emphasize on the importance of mental health issues among orthopaedic surgeons. The association between gender, relationship status, and parenthood with depressive symptoms shows the importance of tailored interventions targeting vulnerable subgroups, such as female surgeons and those living alone. To mitigate the impact of stress and depression on orthopedic surgeons, healthcare institutions should implement comprehensive mental health support programs that offer resources for stress management, counseling services, and peer support groups. It would also be important to encourage a culture of open communication and destigmatizing mental health issues within the medical community can encourage early intervention and promote overall well-being among surgeons.

## Data Availability

All data generated or analyzed during this study are included in this published article.
